# Correlations between Prenatal Exposure to Perfluorinated Chemicals and Reduced Fetal Growth

**DOI:** 10.1289/ehp.11681

**Published:** 2008-11-04

**Authors:** Noriaki Washino, Yasuaki Saijo, Seiko Sasaki, Shizue Kato, Susumu Ban, Kanae Konishi, Rie Ito, Ayako Nakata, Yusuke Iwasaki, Koichi Saito, Hiroyuki Nakazawa, Reiko Kishi

**Affiliations:** 1 Department of Public Health, Hokkaido University Graduate School of Medicine, Sapporo, Japan;; 2 Department of Health Science, Asahikawa Medical College, Asahikawa, Japan;; 3 Department of Analytical Chemistry, Faculty of Pharmaceutical Sciences, Hoshi University, Tokyo, Japan

**Keywords:** birth weight, chest circumference, fetal growth, head circumference, length, perfluorinated chemicals, perfluorooctane sulfonate, perfluorooctanoate, prenatal exposure

## Abstract

**Background:**

Perfluorooctane sulfonate (PFOS) and perfluorooctanoate (PFOA) are man-made, ubiquitous, and persistent contaminants in the environment, wildlife, and humans. Although recent studies have shown that these chemicals interfere with fetal growth in humans, the results are inconsistent.

**Objectives:**

Our goal was to investigate the correlation between relatively low levels of PFOS and PFOA in maternal serum and birth weight and birth size.

**Methods:**

We conducted a hospital-based prospective cohort study between July 2002 and October 2005 in Sapporo, Japan. A total of 428 women and their infants were involved in the study. We obtained characteristics of the mothers and infants from self-administered questionnaire surveys and from medical records. We analyzed maternal serum samples for PFOS and PFOA by liquid chromatography–tandem mass spectrometry (LC/MS/MS).

**Results:**

After adjusting for confounding factors, PFOS levels negatively correlated with birth weight [per log_10_ unit: β = −148.8 g; 95% confidence interval (CI), −297.0 to −0.5 g]. In addition, analyses stratified by sex revealed that PFOS levels negatively correlated with birth weight only in female infants (per log_10_ unit: β = −269.4 g; 95% CI, −465.7 to −73.0 g). However, we observed no correlation between PFOA levels and birth weight.

**Conclusion:**

Our results indicate that *in utero* exposure to relatively low levels of PFOS was negatively correlated with birth weight.

Perfluorinated chemicals, which have been manufactured for > 50 years, have been used in a wide range of industrial and consumer products. Perfluorooctane sulfonate (PFOS) and perfluorooctanoate (PFOA), which are representative of perfluorinated chemicals, have recently been found to be widespread contaminants in the environment, wildlife, and humans (Butenhoff et al. 2006; Key et al. 1997; Lau et al. 2007; Renner 2001). The worldwide distribution of PFOS and PFOA is recognized as a severe problem because of their resistance to further degradation in the environment.

PFOS and PFOA contamination in human blood has been reported in various countries (Butenhoff et al. 2006; Calafat et al. 2007; Harada et al. 2007; Kannan et al. 2004; Lau et al. 2007; Midasch et al. 2006). Maternal serum PFOS and PFOA levels measured in our previous study (Inoue et al. 2004a) were relatively low compared with most levels in previous reports (Butenhoff et al. 2006; Calafat et al. 2007; Harada et al. 2007; Kannan et al. 2004; Lau et al. 2007; Midasch et al. 2006).

Exposure of pregnant rats and mice to PFOS led to a reduction in birth weight (Grasty et al. 2003, 2005; Lau et al. 2003; Luebker et al. 2005a, 2005b; Thibodeaux et al. 2003), and a similar result was obtained when pregnant rats and mice were exposed to PFOA (Abbott et al. 2007; Butenhoff et al. 2004; Lau et al. 2006; Wolf et al. 2007). Interference of lipid metabolism (Kennedy et al. 2004; Loveless et al. 2006; Xie et al. 2003) and alterations in thyroid hormone homeostasis (Lau et al. 2003; Luebker et al. 2005b; Martin et al. 2007; Thibodeaux et al. 2003) have been suggested as possible mechanisms of fetal growth restriction. Currently, however, the mechanism behind any correlation between PFOS and PFOA and fetal growth restriction is not clearly understood.

Human studies have shown no substantial changes in hematologic, lipid, hepatic, thyroid, or urinary characteristics in populations exposed occupationally to perfluorinated chemicals (Olsen et al. 1999, 2003). PFOS and PFOA were detected in nearly 100% of umbilical cord sera in 299 samples (Apelberg et al. 2007a), indicating that human fetuses are exposed to these chemicals. Our previous study and another study have shown the placental permeability of PFOS and PFOA (Inoue et al. 2004a; Midasch et al. 2007). Because fetuses might be more vulnerable than adults to the potential harmful effects of chemicals, exposure assessment studies for perfluorinated chemicals in human fetuses are urgently needed. One occupational study found no correlation between high occupational exposure to PFOS before the end of pregnancy and maternally reported birth weight among 439 singleton live births (Grice et al. 2007). Meanwhile, two reports investigated the correlations between prenatal nonoccupational PFOS and PFOA exposure and reduced fetal growth. One study showed a correlation between PFOS and PFOA levels in cord serum and reduced values for birth weight, ponderal index, and head circumference among 293 women and their infants. The median concentration for PFOA was 1.6 ng/mL and for PFOS was 5 ng/mL (Apelberg et al. 2007b). Another study showed that maternal plasma PFOA levels correlated with reduced birth weight among 1,400 women and their infants. PFOS and PFOA levels in maternal plasma were, on average, 35.3 and 5.6 ng/mL, respectively (Fei et al. 2007).

Given that few reports have actually suggested a correlation between prenatal non-occupational PFOS and PFOA exposure and reduced fetal growth, further research is needed to clarify this potential relationship. The aim of the present study was to investigate the relationship between relatively low levels of PFOS and PFOA in maternal serum and birth weight and birth size, including length, chest circumference, and head circumference.

## Materials and Methods

### Study population

We performed a prospective cohort study between July 2002 and October 2005 at the Sapporo Toho Hospital in Hokkaido, Japan (Hokkaido Study on Environment and Children’s Health). Study subjects were women who enrolled at 23–35 weeks of gestation during a routine gynecologic checkup and delivered at the Sapporo Toho Hospital. All subjects were native Japanese and residents of Sapporo or surrounding areas. Of 1,796 potentially eligible women, 514 agreed to participate (a participation rate of 29%). Among the 514 pregnant women who composed the initial cohort, 10 were excluded because of miscarriage, stillbirth, relocation, or voluntary withdrawal from the study before follow-up.

The subjects completed a self-administered questionnaire survey after the second trimester of pregnancy. The questionnaire included information related to dietary habits, smoking status, alcohol intake, caffeine intake, household income, and educational level. We obtained information on dietary habits during pregnancy, such as intake of eggs, milk and its by-products, carbohydrates, fish, and meat, from responses on the food frequency questionnaire, divided into five categories: rarely/never, one to two times per month, one to two times per week, three to four times per week, or every day. In this study, we based maternal smoking status on maternal self-reporting and categorized it into two groups: nonsmokers [those who did not smoke throughout pregnancy or quit smoking at the beginning of pregnancy (first trimester)] and smokers (those who continued to smoke during pregnancy, which included women who quit after the second trimester).

For estimating alcohol intake, we used the modified self-administered questionnaire described previously by Nagata et al. (1997). We calculated average daily alcohol consumption during pregnancy in grams of ethanol for regular drinkers on the basis of beverage type, frequency of alcoholic beverage consumption, and the amount consumed per occasion during pregnancy. The questionnaire contained queries on the intake of alcoholic beverages popular in Japan, including sake (rice wine), shochu (a type of brandy), beer, light beer, wine, and other kinds of alcohol drinks (e.g., whiskey, brandy, gin, vodka, cocktails, rum). Frequency of alcohol intake during pregnancy was classified into nine categories: rarely/never, once per month, two to three times per month, once per week, two to three times per week, four to six times per week, once per day, two to three times per day, or more than four times per day. The quantity per occasion during pregnancy for sake and shochu was classified into four categories: ≤1 go, 2 go, 3 go, and ≥ 4 go (in Japan, the go is the most commonly used measure of sake and shochu consumption; 1 go of sake and shochu, equivalent to 180 mL, contains approximately 22 g and 37 g ethanol, respectively). The quantity per occasion during pregnancy for beer and light beer was classified into four categories: one can of beer or less, one bottled beer, two to three bottled beers, four or more bottled beers. One can of beer and light beer contains approximately 14 g and 10 g ethanol, respectively, and one bottle of beer and light beer contains approximately 25 g and 15 g ethanol, respectively. The quantity per occasion during pregnancy for wine and the other kinds of alcohol drinks was classified into four categories: one glass or less, two glasses, three glasses, four or more glasses. One glass of wine and the other kinds of alcohol drinks contains approximately 11 g and 21 g ethanol, respectively. We calculated the amount of ethanol consumed per day as the amount consumed per occasion multiplied by the ethanol content for each beverage type multiplied by the frequency of intake per day. In the present study, we used total alcohol intake from all beverages as the “exposure.”

For estimating caffeine intake, we used the modified self-administered questionnaire described previously by Nagata et al. (1998). We calculated average daily caffeine consumption during pregnancy in milligrams of caffeine for regular drinkers on the basis of beverage type, frequency of caffeinated beverage consumption, and the amount consumed per occasion during pregnancy. The questionnaire contained queries on the intake of caffeinated beverages popular in Japan, including coffee [one cup of instant coffee (60 mg caffeine), regular coffee (50 mg), decaffeinated coffee (0 mg), and canned coffee (50 mg)], tea [one cup of black tea (60 mg), oolong tea (30 mg), powdered green tea (200 mg), Gyokuro (green tea of the highest quality; 100 mg), and other kinds of tea such as natural leaf tea, coarse green tea, brown rice tea, and roasted green tea (30 mg)], one cup of cocoa (5 mg), one glass of cola (60 mg), and one bottle of caffeinated health drink (50 mg). Frequency of intake during pregnancy was divided into five categories: rarely/never, once or twice per month, once or twice per week, three to four times per week, or every day. The quantity per occasion for caffeinated beverage during pregnancy was classified into four categories; one cup (glass or bottle) or less, two cups (glasses or bottles), three cups (glasses or bottles), and four cups or more (glasses or bottles). We calculated the caffeine amount consumed per day as the amount consumed per occasion multiplied by the caffeine content of beverage type multiplied by the frequency of intake per day. In the present study, we used total caffeine intake from all beverages as the “exposure.”

Annual household income based on maternal self-reporting was divided into five categories: < 3 million yen, 3–5 million yen, 5–7 million yen, 7–10 million yen, or ≥ 10 million yen. Maternal educational level based on maternal self-reporting was divided into four categories: 9 years, 10–12 years, 13–16 years, or ≥ 17 years. We obtained the information for exclusion criteria and potential confounders of the mothers and their infants—prepregnancy body mass index (BMI), pregnancy complications, gestational age, infant sex, parity, infant disease, birth weight, and birth size—from medical records written by gynecologists. This study was conducted with written informed consent from all subjects and was approved by the institutional ethical board for epidemiologic studies at the Hokkaido University Graduate School of Medicine.

### Exposure assessment

A 40-mL blood sample was taken from the maternal peripheral vein after the second trimester of pregnancy. When this was not possible because of anemia of the mother, we took a blood sample after delivery. All samples were stored at −80°C until analysis. The analytical detection method used is the variation of a method published previously (Nakata et al. 2005a, 2005b), and the methods for developing this analytical approach are also available elsewhere (Inoue et al. 2004a, 2004b).

We obtained PFOS [molecular weight (MW), 538.23 Da; 98% purity] and PFOA (MW, 414.07 Da; > 90% purity) from Fluka Chemie AG (Buchs, Switzerland). We purchased perfluoroheptanoic acid (PFHpA, 99%), which we used as an internal standard, from Sigma Chemical Co. (St. Louis, MO, USA). We purchased acetonitrile and methanol for high-performance liquid chromatography and analytical-grade ammonium acetate and acetic acid from Wako Pure Chemical Industries, Ltd. (Osaka, Japan). The water purification system was a Milli-Q gradient A 10 (185 nm ultraviolet, ion exchange, and filtration) equipped with an EDS polisher (activated carbon filtration) (Millipore, Bedford, MA, USA).

### Instrumentation and analytical conditions for column-switching liquid chromatography– tandem mass spectrometry coupled with solid-phase extraction

We performed liquid chromatography–tandem mass spectrometry (LC/MS/MS) with a Waters Quattro micro system (Nihon Waters KK, Tokyo, Japan). Separation was achieved on an Inertsil ODS-3 column (2.1 × 50 mm, 5 μm; GL Sciences Inc., Tokyo, Japan) equipped with a Mightysil RP-18 GP precolumn (2.0 × 5 mm, 5 μm; Kanto Chemical Inc., Osaka, Japan). The column oven was maintained at 40°C. The column-switching LC/MS/MS was coupled with an online extraction system consisting of the LC/MS/MS combined with an LC pump (LC-10ADvp pump; Shimadzu, Kyoto, Japan) and a Waters Oasis HLB extraction column (2.1 × 20 mm, 25 μm). After we injected a 50-μL sample with an autosampler, we loaded it onto the extraction column by flowing 50 mM acetate buffer (pH = 4.7):methanol (90:10, vol/vol) at a flow rate of 1.0 mL/min. The valve was switched 5 min after sample injection. We eluted the sample through a back-flashing extraction column to the analytical column and introduced it into the MS/MS apparatus. We carried out separation using a mobile phase of 1.0 mM ammonium acetate/acetonitrile (vol/vol) at a flow rate of 0.2 mL/min. The gradient profile was as follows: linear increase from 45% to 85% acetonitrile solution for 5–12 min, then hold at 85% for 3 min. The conditions for MS/MS were as follows: Desolvation and source temperatures were set at 350°C and 100°C, respectively, and the capillary was held at a potential of 600 V relative to the counterelectrode in the negative-ion mode for all compounds. Cone and desolvation gas flow rates were 50 and 350 L/hr, respectively. Cone and collision voltages were 60 and 65 V for PFOS, 30 and 18 V for PFOA, and 28 and 18 V for PFHpA, respectively. When we tested the recoveries using human plasma samples, the recoveries for spiked 5 and 50 ng/mL PFOS were 99.3% [coefficient of variation (CV), 3.0%] and 97.5% (CV, 6.3%), respectively (*n* = 6), and the recoveries for spiked 1 and 10 ng/mL PFOA were 100.0% (CV, 8.9%) and 97.3% (CV, 4.8%), respectively (Nakata et al. 2005a, 2005b). In addition, we further verified reliability of our method through participation in the First International Laboratory Calibration Study coordinated by Animal Sciences Group-The Netherlands Institute for Fisheries Research (ASG-RIVO) and Örebro University (Lindström et al. 2005). Our laboratory reported acceptable results. Thus, we judged our results to be accurate and precise.

### Sample preparation

We mixed human plasma samples (0.1 mL) with 0.2 mL internal standard solution containing acetonitrile, centrifuged the mix at 1,450 × g for 10 min, and transferred the supernatant to a polypro-pylene tube. We subjected the aliquot of the filtered sample solution to column-switching LC/MS/MS.

As a result of loss to follow-up, lack of serum specimen, and laboratory capacity, we ultimately measured the concentrations of perfluorinated chemicals in 447 maternal sera samples. We measured the concentrations of 7 polychlorinated dibenzo-*p*-dioxins, 10 polychlorinated dibenzofurans, and 4 non-*ortho*-polychlorinated biphenyls (PCBs) using high-resolution gas chromatography/high resolution mass spectrometry (HRGC/HRMS) equipped with a solvent cut large-volume (SCLV) injection system (SGE Ltd., Victoria, Australia) and we also measured 8 mono-*ortho*-PCBs and 58 non-dioxin-like PCB congeners in maternal blood (n = 370) using HRGC/HRMS at Fukuoka Institute of Health and Environmental Sciences. The analytical method is available elsewhere (Todaka et al. 2007, 2008).

### Data analysis

For analysis of maternal PFOS and PFOA levels and correlations with birth weight and birth size, we excluded subjects having maternal pregnancy-induced hypertension, diabetes mellitus, fetal heart failure, and twins. Ultimately, we included in the analysis 428 mother–infant pairs for whom we had measured PFOS and PFOA concentrations.

We analyzed correlations between PFOS and PFOA concentrations and characteristics of mothers and infants by the Spearman correlation test, Mann–Whitney *U*-test, and Kruskal–Wallis test and analyzed correlations between birth weight and birth size and characteristics of mothers and infants by the Student’s *t*-test and Pearson’s correlation test. Finally, we performed multiple regression analyses to determine correlations between PFOS and PFOA levels and birth weight and birth size. Because of the skewed distributions, we treated the levels of PFOS and PFOA in maternal serum as a continuous variable on a log_10_ scale. For subjects with a level less than the detection limit, we used a value equal to half of the detection limit. The fully adjusted model used multiple regression analysis adjusted for maternal age, maternal educational level, smoking status during pregnancy, maternal BMI, parity, infant sex, and gestational age. For head circumference, we included delivery mode (vaginal delivery vs. cesarean section) in the fully adjusted model as a potential predictor of head circumference. When we examined the levels of PFOS and PFOA in samples taken at different times (during pregnancy and after delivery) by the Mann–Whitney *U*-test, we found significant differences in the levels of PFOS (*p* < 0.001) and PFOA (*p* < 0.001). Thus, we also adjusted the blood sampling time in the fully adjusted model. We performed all statistical analyses using SPSS for Windows, version 13.0J (Japanese version; SPSS, Inc., Chicago, IL, USA). Results were considered statistically significant if *p* < 0.05.

## Results

Table 1 shows maternal serum PFOS and PFOA concentrations in relation to characteristics of mothers and infants. In total, we included 428 mother–infant pairs in the study. The mean (± SD) maternal age was 30.5 ± 4.8 years. Maternal educational level was divided into two categories, ≤12 years and ≥ 13 years, because the numbers of subjects in the “9 years” and “≥ 17 years” categories were only 11 (2.6%) and 6 (1.4%), respectively. The numbers of subjects in the two categories, ≤ 12 years and ≥ 13 years, were 192 (44.9%) and 236 (55.1%), respectively. For annual income, the number of subjects in the “≥ 10 million yen” category was only 5 (1.2%), so we included these in the “≥ 7 million yen” category. The numbers of subjects in the four categories, < 3 million yen, 3–5 million yen, 5–7 million yen, and ≥ 7 million yen, were 86 (20.2%), 210 (49.4%), 86 (20.2%), and 43 (10.1%), respectively. The number of subjects in the category “maternal smoking during pregnancy,” which included women who quit smoking after the second trimester, was 94 (22.0%), and the number in the category “maternal nonsmoking during pregnancy,” which included women who quit in the first trimester, was 334 (78.0%). The numbers of subjects in the category “blood sampling during pregnancy” and “blood sampling after delivery” were 310 (72.4%) and 118 (27.6%), respectively. There were 198 male infants (46.3%) and 230 female infants (53.7%). The mean (± SD) gestational age was 275.5 ± 9.9 days. We observed statistically significant differences (*p* < 0.05) in median PFOS concentrations by age, smoking status, blood sampling period, type of delivery, gestational age, and parity, and we observed differences in median PFOA concentrations by caffeine intake, blood sampling period, gestational age, infant sex, and parity. Table 2 shows birth outcomes of study subjects. The mean (± SD) birth weight was 3058.1 ± 376.3 g.

**Figure 1:**
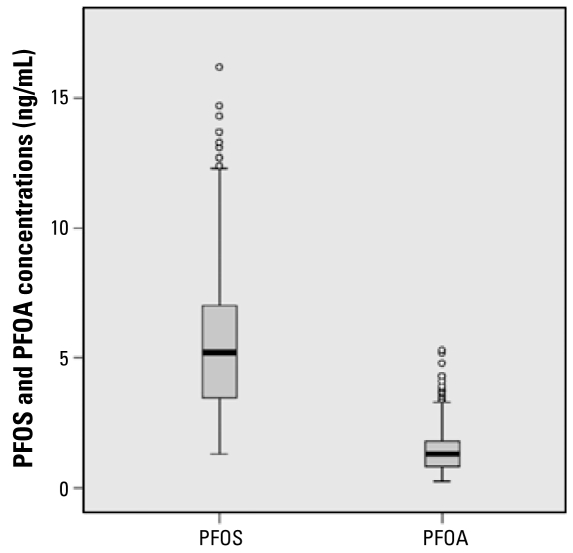
Box and whisker plots of PFOS and PFOA concentrations in maternal serum. Horizontal lines inside boxes indicate the median, boxes represent the interquartile range, whiskers indicate the most extreme data points < 1.5 times the interquartile range from the ends of the box, and circles indicate outliers. Subjects with PFOA level < 0.5 ng/mL are noted as 0.25 ng/mL.

Table 3 presents PFOS and PFOA concentrations included in the analysis (*n* = 428), and Figure 1 shows box and whisker plots of PFOS and PFOA concentrations. Both detection limits were 0.5 ng/mL. We detected PFOS in every maternal serum sample and PFOA in 92.8% of samples. PFOA level was below the detection limit in 31 (7.2%) samples. Concentrations ranged from 1.3 to 16.2 ng/mL for PFOS and from below the detection limit to 5.3 ng/mL for PFOA. Concentrations of PFOS and PFOA in maternal serum were highly correlated (Spearman rank correlation coefficient, 0.242; *p* < 0.01).

Table 4 presents relationships between birth weight and birth size and subject characteristics; we compared the data using the Student’s *t*-test and Pearson’s correlation test. The mean birth weight, chest circumference, and head circumference were significantly lower in the smoker group compared with nonsmoker group (*p* < 0.05). Gestational age (days) had the greatest positive impact on infant birth weight and birth size (all *p* < 0.001). Maternal prepregnancy BMI positively correlated with birth weight (*p* < 0.05). Length and head circumference in male infants were significantly greater compared with female infants (*p* = 0.001 and *p* < 0.001, respectively). Head circumference for first infants was significantly smaller than for subsequent infants (*p* < 0.05).

**Figure 2:**
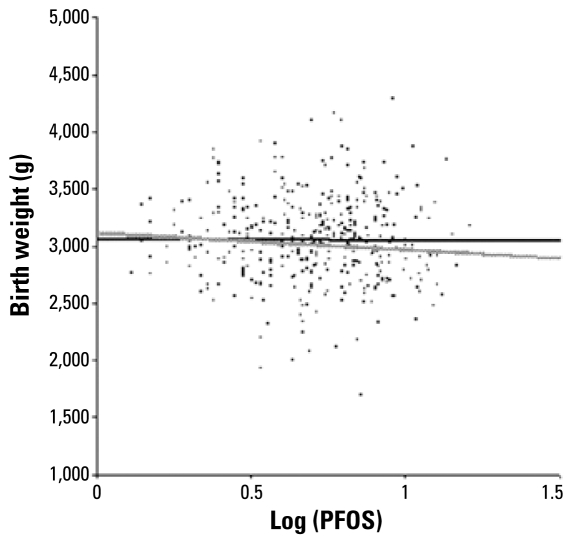
Birth weight in both male and female infants and log_10_ maternal PFOS concentrations, before and after adjustment for potential confounders. The black line denotes the predicted fit from a simple linear regression model (birth weight = 3068.6–15.1 log_10_ PFOS); the gray line denotes the predicted fit from the fully adjusted multivariate regression model (birth weight = 3127.3–148.8 log_10_ PFOS). Table 5 presents corresponding regression coefficients.

Table 5 shows univariate and multivariate regression model results for birth weight and birth size on log_10_-transformed PFOS and PFOA concentrations. Multivariate models of birth weight and birth size are adjusted for gestational age only or fully adjusted; the fully adjusted model includes risk factors correlated with birth weight and birth size at *p*-values < 0.05, risk factors known to be correlated with birth weight and birth size from previous reports, and blood sampling period (i.e., maternal age, maternal educational level, maternal smoking status during pregnancy, maternal BMI, parity, infant sex, gestational age, blood sampling period; delivery mode was included for head circumference). In the crude model, we found no significant correlations between PFOS and PFOA levels and birth weight and birth size. In the model adjusted only for gestational age, a log_10_-unit increase in PFOS and PFOA levels correlated with a decrease in mean birth weight of 148.7 g [95% confidence interval (CI), 291.2 to 6.2 g] for PFOS and 119.1 g (95% CI, 228.8 to 9.3 g) for PFOA. Further adjustment for parity resulted in a loss of the significant negative correlation between PFOA levels and birth weight, so the fully adjusted model showed no significant negative correlation between PFOA levels and birth weight. In contrast, a log_10_-unit increase in PFOS levels correlated with a decrease in mean birth weight of 148.8 g (95% CI, 297.0 to 0.5 g) for PFOS in the fully adjusted model. Figure 2 plots the predicted values from selected regression models in male and female infants across the full range of log_10_-transformed maternal PFOS concentrations.

Table 6 shows the results of multiple regression analyses between maternal PFOS and PFOA levels and birth weight and birth size in male infants. In the model adjusted only for gestational age, a log_10_-unit increase in PFOA levels correlated with a decrease in mean birth weight of 166.4 g (95% CI, 328.9 to 4.0 g). Further adjustment for parity resulted in a loss of significant negative correlation between PFOA levels and birth weight in male infants, so the fully adjusted model shows no significant negative correlation between PFOA levels and birth weight in male infants. In the crude model, a log_10_-unit increase in PFOS levels correlated with an increase in mean length of 1.230 cm (95% CI, 0.075 to 2.386 cm). However, after adjustment for gestational age, the significant positive correlation disappeared.

**Figure 3:**
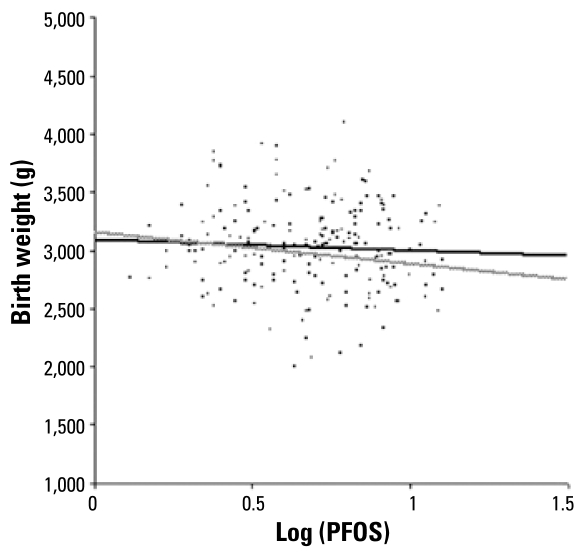
Birth weight and log_10_ PFOS concentrations in females, before and after adjustment for potential confounders. The black line denotes the predicted fit from a simple linear regression model (birth weight = 3087.8–90.7 log_10_ PFOS); the gray line denotes the predicted fit from the fully adjusted multivariate regression model (birth weight = 3163.4–269.4 log_10_ PFOS). Table 7 presents corresponding regression coefficients.

Table 7 shows the results of multiple regression analyses between maternal PFOS and PFOA levels and birth weight and birth size in female infants. In the crude model, we observed no significant correlations between PFOS levels and birth weight. In the model adjusted only for gestational age, a log_10_-unit increase in PFOS levels correlated with a decrease in the mean birth weight of 237.1 g (95% CI, 425.0 to 49.1 g). In the fully adjusted model, a log_10_-unit increase in PFOS levels correlated with a decrease in mean birth weight of 269.4 g (95% CI, 465.7 to 73.0 g). Figure 3 plots the predicted values from selected regression models in female infants across the full range of log_10_- transformed PFOS concentrations. We observed marginally significant negative correlations between PFOS levels and length and chest circumference in the fully adjusted model.

Among the subjects analyzed in this study (*n* = 428), we measured the levels of total dioxins in maternal blood [sum of 7 polychlorinated dibenzo-*p*-dioxins, 10 polychlorinated dibenzofurans, and 12 dioxin-like PCBs (four non-*ortho* PCBs and eight mono-*ortho* PCBs)] (*n* = 318) and the levels of total PCBs (sum of 70 PCB congeners (58 non-dioxin-like PCB congeners and 12 dioxin-like PCBs) in maternal blood (*n* = 318) (data not shown). Total dioxins levels positively correlated with PFOS and PFOA levels [Spearman rank correlation coefficients, 0.149 (*p* = 0.008) and 0.162 (*p* = 0.004), respectively]. Furthermore, total PCB levels positively correlated with PFOA levels (Spearman rank correlation coefficient, 0.136; *p* = 0.015) but not with PFOS levels (Spearman rank correlation coefficient, 0.089; *p* = 0.111). However, the fully adjusted regression coefficients between PFOS and PFOA levels and birth weight also changed little when we adjusted for total dioxins and total PCB levels (data not shown).

## Discussion

Our results indicate that *in utero* exposure to relatively low levels of PFOS negatively correlated with birth weight, but PFOA levels did not correlate with birth weight. Two previous studies suggested that prenatal nonoccupational PFOS and PFOA exposure correlated with reduced fetal growth (Apelberg et al. 2007b; Fei et al. 2007). One study showed a significant negative correlation between maternal plasma PFOA levels, but not PFOS levels, and birth weight in a prospective cohort study among 1,400 women and their infants (Fei et al. 2007), and the other study showed a negative correlation between cord serum PFOS and PFOA levels and birth weight in a cross-sectional study among 293 women and their infants (Apelberg et al. 2007b). Our results are inconsistent with these two reports. Differences in blood sample types, sampling periods, and laboratories may have affected the results, but these differences are unlikely to account entirely for the inconsistency of the findings. Differences in confounder adjustment could be an alternative explanation. Fei et al. (2007) adjusted for quadratic gestational age, and Apelberg et al. (2007b) adjusted for maternal height and net weight gain during pregnancy, both of which are confounding factors not considered in our fully adjusted model. However, our regression coefficients did not change markedly when we adjusted for these variables. On the other hand, differences in PFOS and PFOA concentrations also could be an alternative explanation. Fei et al. (2007) reported that PFOS and PFOA levels in maternal plasma were, on average, 35.3 (range, 6.4 to 106.7 ng/mL) and 5.6 ng/mL (range, < 1.0 to 41.5 ng/mL), respectively. Serum-to-plasma ratios for PFOS and PFOA have been reported as 1:1 (Ehresman et al. 2007). Our cohort study showed average PFOS and PFOA levels in maternal serum to be 5.6 ng/mL (range, 1.3 to 16.2 ng/mL) and 1.4 ng/mL (range, < 0.5 to 5.3 ng/mL), respectively. Although the levels of PFOS and PFOA measured in our cohort are lower, we observed a negative correlation between only PFOS levels and birth weight. Apelberg et al. (2007b) reported that the median cord serum PFOS and PFOA levels were 5 ng/mL (range, < 0.2 to 34.8 ng/mL) and 1.6 ng/mL (range, 0.3 to 7.1 ng/mL), respectively. The mean ratio of PFOS and PFOA concentrations in maternal plasma to that in cord plasma has been reported as 0.60 and 1.26, respectively (Midasch et al. 2007). Additionally, our previous study showed a mean ratio of 0.32 for PFOS concentrations in maternal serum to that in cord serum (Inoue et al. 2004a). In our present cohort study, we determined median levels of PFOS and PFOA in maternal serum to be 5.2 ng/mL (range, 1.3 to 16.2 ng/mL) and 1.3 ng/mL (range, < 0.5 to 5.3 ng/mL), respectively. Our PFOS levels might be lower and PFOA levels might be nearly the same as those reported by Apelberg et al. (2007b), but we did not find a correlation between PFOA levels and reduced birth weight. We cannot fully explain the inconsistencies between our results and the two previous reports with respect to differences in PFOS and PFOA concentrations. At present, we are currently establishing a large multihospital-based birth cohort in Hokkaido prefecture. Using data from this large cohort, we plan to investigate the relationship between perfluorinated chemicals and reduced birth weight with a larger number of mother–infant pairs in a further study.

PFOS and PFOA levels in our study samples from residents of Sapporo City, Hokkaido, Japan, were relatively lower compared with studies in Europe, United States, and other domestic areas in Japan (Butenhoff et al. 2006; Calafat et al. 2007; Harada et al. 2007; Kannan et al. 2004; Lau et al. 2007; Midasch et al. 2006). Despite relatively lower PFOS levels, we found a negative correlation between PFOS levels and birth weight.

One recent occupational study showed no correlation between high occupational exposure to PFOS before the end of pregnancy and maternally reported birth weight (Grice et al. 2007). However, that study did not adjust for the length of gestation, which affects birth weight. Therefore, the inconsistency between that study and our study might be due to inadequate adjustment for gestational period.

Our results show that female infants may be more vulnerable to PFOS than are male infants. However, Fei et al. (2007) reported no correlation between PFOS levels and birth weight in neonates after stratification by sex. We were unable to infer a mechanism for the sex difference in the toxicity of PFOS. It is possible that decreasing the sample size by stratifying our groups by sex affected our results. Thus, further studies are needed to clarify any sex-related affects of *in utero* PFOS exposure on birth weight.

In our study, *in utero* exposure to PFOS negatively correlated with birth weight, which is consistent with studies in laboratory animals (Abbott et al. 2007; Butenhoff et al. 2004; Grasty et al. 2003, 2005; Lau et al. 2003, 2006; Luebker et al. 2005a, 2005b; Thibodeaux et al. 2003; Wolf et al. 2007). We observed an inverse correlation between PFOS levels and birth weight, although the maternal serum concentrations were orders of magnitude lower than those at which developmental effects have been observed in the animal toxicity studies. Luebker et al. (2005b) estimated a lower bound of the benchmark dose correlated with a 5% change in response for PFOS and birth weight in rats of 0.39 mg/kg/day, equivalent to rat fetal serum concentrations of 34,000 ng/mL. Humans might be more sensitive to PFOS than are rats and mice; this might be attributable partly to differences between humans and rats/mice with respect to half-life for elimination of PFOS—the half-life for rats is approximately 100 days (Johnson et al. 1979), whereas that for humans is approximately 5 years (Olsen et al. 2005). At present, the toxicokinetic data and the mechanisms related to PFOS toxicity in humans are insufficient to explain the inter-species differences in sensitivity to PFOS.

Concentrations of PFOS and PFOA decreased with increasing parity, which is consistent with two previous reports (Apelberg et al. 2007b; Fei et al. 2007). This might be attributable to placental transfer of perfluorinated chemicals to the fetus (Inoue et al. 2004a; Midasch et al. 2007) and transfer to the infant through breast-feeding (Kärrman et al. 2007; So et al. 2006). PFOS and PFOA levels sampled during pregnancy were significantly higher than PFOS and PFOA levels sampled after delivery. This might be attributable to blood volume expansion and decreased serum albumin concentration during pregnancy (Swanson and King 1983; Thibodeaux et al. 2003), changes in perfluorinated chemical disposition and pharmacokinetics during pregnancy (Frederiksen 2001), and placental transfer of perfluorinated chemicals to the fetus (Inoue et al. 2004a; Midasch et al. 2007). In our study, PFOS levels decreased with increasing maternal age, which is consistent with one report (Fei et al. 2007), and PFOS levels in smokers are lower than in nonsmokers, which is consistent with another report (Apelberg et al. 2007b). After adjusting parity and blood sampling period in multiple regression analysis, the correlation between log_10_-transformed PFOS levels and maternal age and smoking status remained significant (*p* = 0.024 and *p* = 0.007, respectively). However, after adjusting for parity, blood sampling period, smoking status during pregnancy, and maternal age in a multiple regression analysis, the significant correlations between log_10_-transformed PFOS levels and type of delivery and gestational age disappeared (*p* = 0.059 and *p* = 0.317, respectively). Thus, the correlations between log_10_-transformed PFOS levels and type of delivery and gestational age might have been confounded by parity, blood sampling period, smoking status during pregnancy, and maternal age. Further, after adjusting parity and blood sampling period in a multiple regression analysis, the significant correlations between log_10_-transformed PFOA levels and caffeine intake and gestational age also disappeared (*p* = 0.114 and *p* = 0.584, respectively). Thus, the significant correlations between log_10_-transformed PFOA levels and caffeine intake and gestational age might have been confounded by parity and blood sampling period. On the other hand, the correlations between log_10_-transformed PFOA levels and sex were not significant after adjustment for parity and blood sampling period (*p* = 0.071). In addition, besides the covariates in our final model, we evaluated the caffeine intake as a possible confounding factor; this adjustment did not materially change the fully adjusted regression coefficients between PFOS and PFOA levels and birth weight (data not shown).

Our study constitutes a prospective cohort study, which minimizes recall bias. We collected prenatal information on the mother and child, such as disease history, pregnancy conditions, and birth weight and birth size, from medical records written by gynecologists, not from maternally reported information, adding data reliability. Besides the covariates in our final model, we evaluated the frequency of food intake (eggs, milk and its by-products, carbohydrates, fish, and meat), season of blood sampling, and season of birth as possible confounding factors. These adjustments did not materially change the fully adjusted regression coefficients between PFOS and PFOA levels and birth weight (data not shown). This study has some limitations. First, our sample size was relatively small compared with the study by Fei et al. (2007). With a larger sample size, the correlations between maternal PFOS levels and chest circumference and length may be clearer. Second, selection bias may have occurred because we based our study on a cohort from one area hospital, which treated pregnant women in Sapporo and the surrounding areas. In addition, the participation rate in our cohort study is low (29%), which also may have caused selection bias. Third, anthropometric measurements (length, chest, and head circumferences) may have errors due to length and chest and head molding.

This study shows a correlation between maternal PFOS levels and reduced fetal growth. PFOS cannot be degraded by the environment, and therefore we must evaluate its potential toxicity in humans, especially children. Other than birth weight, animal studies have shown effects of PFOS and PFOA on numerous organ systems, thyroid function, endocrine system, immune function, and congenital anomalies (Chang et al. 2008; Luebker et al. 2005b; Peden-Adams et al. 2007; Seacat et al. 2002; Thibodeaux et al. 2003). Little research has been conducted on the toxicity of PFOS and PFOA in human fetuses. Thus, our future studies will investigate whether PFOS and PFOA levels correlate with thyroid hormone levels, immune function, and neural development in human fetuses and infants.

## Correction

In the third-to-last paragraph, the Spearman rank correlation coefficients and *p*-values were incorrect in the manuscript originally published online; they have been corrected here.

## Figures and Tables

**Table 1:** Maternal serum PFOS and PFOA concentrations [median (interquartile range)] in relation to maternal and infant characteristics (*n* = 428).

Characteristic	No. (%)	PFOS	PFOA
Maternal characteristics			
Age (years)*	30.5 ± 4.8^a^	*r* = −0.173	*r* = −0.095
Educational level (years)			
≤ 12	192 (44.9)	4.9 (3.6–6.6)	1.2 (0.8–1.8)
≥ 13	236 (55.1)	5.5 (3.4–7.4)	1.3 (0.8–1.8)
Annual household income (million yen)^b^			
< 3	86 (20.2)	5.5 (3.8–7.8)	1.4 (0.8–2.0)
3–5	210 (49.4)	5.0 (3.4–6.8)	1.2 (0.8–1.6)
5–7	86 (20.2)	5.5 (3.5–7.0)	1.3 (0.9–1.8)
≥ 7	43 (10.1)	5.8 (3.4–7.2)	1.5 (0.9–2.4)
Maternal smoking status during pregnancy*			
Nonsmoking	334 (78.0)	5.3 (3.7–7.3)	1.3 (0.8–1.8)
Smoking	94 (22.0)	4.7 (3.0–6.5)	1.2 (0.8–1.6)
Alcohol intake during pregnancy			
No	299 (69.9)	5.2 (3.5–6.9)	1.3 (0.8–1.8)
Yes	129 (30.1)	5.3 (3.4–7.3)	1.3 (0.8–1.8)
Alcohol intake among drinkers during pregnancy (g/day)	1.4 (0.4–152.0)^c^	*r* = 0.024	*r* = −0.033
Caffeine intake during pregnancy (mg/day)**	124.0 (2.0–1242.5)^c^	*r* = −0.064	*r* = −0.131
Prepregnancy BMI (kg/m^2^ )	21.1 ± 3.1^a^	*r* = −0.079	*r* = −0.091
Blood sampling period*^,^**			
During pregnancy	310 (72.4)	5.6 (4.1–7.5)	1.4 (0.9–2.0)
After delivery	118 (27.6)	3.8 (2.5–5.6)	1.0 (0.6–1.5)
Type of delivery*			
Vaginal	343 (80.1)	5.4 (3.8–7.3)	1.3 (0.8–1.8)
Cesarean section	85 (19.9)	4.3 (3.0–6.5)	1.2 (0.8–1.6)

Infant characteristics			
Gestational age (days)*^,^**	275.5 ± 9.9^a^	*r* = 0.130	*r* = 0.107
Sex**			
Male	198 (46.3)	5.2 (3.7–7.0)	1.4 (0.9–1.9)
Female	230 (53.7)	5.2 (3.2–7.1)	1.2 (0.8–1.7)
Parity*^,^**			
0	202 (47.2)	5.8 (4.0–8.0)	1.6 (1.2–2.1)
≥ 1	226 (52.8)	4.8 (3.0–6.4)	0.9 (0.6–1.4)

a Mean ± SD.

b Data for annual income were missing for three mothers.

c Median (minimum–maximum).

*** Statistically significant differences (*p* < 0.05) using the Spearman’s correlation test, Mann–Whitney *U*-test, and Kruskal–Wallis test for PFOS (*) for PFOA (**).

**Table 2:** Birth outcomes for study end points (*n* = 428).

End point	Mean ± SD
Birth weight (g)	3058.1 ± 376.3
Length (cm)	48.1 ± 1.8
Chest circumference (cm)	31.4 ± 1.6
Head circumference (cm)	33.2 ± 1.3

**Table 3:** Concentrations of PFOS and PFOA (ng/mL) in maternal serum (*n* = 428).

	Detection limit^a^	ND (%)	Mean	Minimum	Percentile			Maximum	Geometric mean
					25th	50th	75th		
PFOS	0.5	0 (0)	5.6	1.3	3.4	5.2	7.0	16.2	4.9
PFOA	0.5	31 (7.2)	1.4	ND	0.8	1.3	1.8	5.3	1.2

ND, nondetectable.

a For subjects with a level below the detection limit, we used a value equal to half the detection limit.

**Table 4:** Relationship between birth weight and birth size and subject characteristics [mean ± SD (*n* = 428)].

Characteristic	No.	Birth weight (g)	*p*-Value	Length (cm)	*p*-Value	Chest circumference (cm)	*p*-Value	Head circumference (cm)	*p*-Value
Maternal characteristics									
Age (years)		*r* = −0.041	0.397	*r* = −0.022	0.646	*r* = −0.021	0.665	*r* = 0.051	0.289
Educational level (years)									
≤ 12	192	3028.4 ± 362.0	0.141	47.9 ± 1.8	0.095	31.3 ± 1.5	0.172	33.1 ± 1.2	0.159
≥ 13	236	3082.3 ± 386.6		48.2 ± 1.8		31.5 ± 1.7		33.3 ± 1.3	
Annual household income (million yen)									
< 5	296	3071.3 ± 366.9	0.360	48.1 ± 1.7	0.550	31.4 ± 1.6	0.664	33.2 ± 1.3	0.982
≥ 5	129	3034.9 ± 398.0		48.0 ± 1.9		31.4 ± 1.7		33.2 ± 1.3	
Smoking status during pregnancy									
Nonsmoker	334	3080.0 ± 384.4	0.023*	48.1 ± 1.8	0.162	31.5 ± 1.6	0.036*	33.3 ± 1.3	0.023*
Smoker	94	2980.5 ± 336.4		47.9 ± 1.6		31.1 ± 1.4		33.0 ± 1.1	
Alcohol intake among drinkers during pregnancy (g/day)		*r* = 0.027	0.760	*r* = 0.022	0.809	*r* = 0.057	0.518	*r* = 0.013	0.883
Caffeine intake during pregnancy (mg/day)		*r* = −0.057	0.237	*r* = −0.064	0.185	*r* = −0.041	0.400	*r* = −0.011	0.828
Prepregnancy BMI		*r* = 0.104	0.032*	*r* = 0.043	0.376	*r* = 0.058	0.229	*r* = 0.070	0.151

Infant characteristics									
Sex									
Male	198	3095.8 ± 378.7	0.055	48.4 ± 1.8	0.001*	31.5 ± 1.5	0.255	33.6 ± 1.3	< 0.001*
Female	230	3025.8 ± 372.0		47.8 ± 1.7		31.3 ± 1.7		33.0 ± 1.2	
Gestational age (days)		*r* = 0.500	< 0.001*	*r* = 0.464	< 0.001*	*r* = 0.465	< 0.001*	*r* = 0.218	< 0.001*
Parity									
0	202	3031.4 ± 381.3	0.165	48.1 ± 1.7	0.854	31.3 ± 1.7	0.212	33.1 ± 1.3	0.019*
≥ 1	226	3082.0 ± 371.0		48.1 ± 1.8		31.5 ± 1.5		33.4 ± 1.3	

* Statistically significant, *p*-value < 0.05, Student’s *t*-test, Pearson’s correlation test.

**Table 5:** Regression coefficients (95% CI) between PFOS and PFOA concentrations (ng/mL) in maternal serum and birth weight and birth size (*n* = 428).

Dependent variable	Partial regression coefficient of log_10_ PFOS^a^	*p*-Value	Partial regression coefficient of log_10_ PFOA^a^	*p*-Value
Birth weight (g)				
Crude	−15.1 (−178.3 to 148.1)	0.856	−39.8 (−166.1 to 86.4)	0.536
Adjusted for gestational age	−148.7 (−291.2 to −6.2)	0.041*	−119.1 (−228.8 to −9.3)	0.034*
Fully adjusted^b^	−148.8 (−297.0 to −0.5)	0.049*	−75.1 (−191.8 to 41.6)	0.207

Length (cm)				
Crude	0.488 (−0.283 to 1.258)	0.214	0.216 (−0.381 to 0.813)	0.478
Adjusted for gestational age	−0.086 (−0.779 to 0.607)	0.807	−0.127 (−0.660 to 0.407)	0.641
Fully adjusted^b^	−0.183 (−0.912 to 0.546)	0.622	−0.140 (−0.712 to 0.432)	0.631

Chest circumference (cm)				
Crude	0.138 (−0.553 to 0.829)	0.696	−0.067 (−0.602 to 0.468)	0.806
Adjusted for gestational age	−0.384 (−1.003 to 0.235)	0.224	−0.378 (−0.854 to 0.099)	0.120
Fully adjusted^b^	−0.389 (−1.046 to 0.268)	0.245	−0.194 (−0.710 to 0.322)	0.460

Head circumference (cm)				
Crude	−0.077 (−0.641 to 0.487)	0.788	−0.083 (−0.519 to 0.354)	0.710
Adjusted for gestational age	−0.280 (−0.836 to 0.277)	0.324	−0.202 (−0.631 to 0.227)	0.355
Fully adjusted^b^	−0.204 (−0.781 to 0.372)	0.486	−0.051 (−0.503 to 0.400)	0.823

a Because PFOS and PFOA levels were log_10_-transformed, partial regression coefficients represent the expected change in dependent variables as a result of a 10-fold change in PFOS and PFOA levels.

b Adjusted for maternal age, maternal educational level, smoking status during pregnancy, maternal BMI, parity, infant sex, gestational age, and blood sampling period. For head circumference, adjusted model also includes delivery mode (cesarean section/vaginal).

* Statistically significant, *p*-value < 0.05.

**Table 6:** Regression coefficients (95% CI) between PFOS and PFOA concentrations (ng/mL) in maternal serum and birth weight and birth size in male infants (*n* = 198).

Dependent variable	Partial regression coefficient of log_10_ PFOS^a^	*p*-Value	Partial regression coefficient of log_10_ PFOA^a^	*p*-Value
Birth weight (g)				
Crude	61.7 (−184.8 to 308.2)	0.622	−66.4 (−254.0 to 121.3)	0.486
Adjusted for gestational age	−68.7 (−284.1 to 146.7)	0.530	−166.4 (−328.9 to −4.0)	0.045*
Fully adjusted^b^	12.1 (−217.7 to 242.0)	0.917	−68.1 (−246.2 to 110.0)	0.452

Length (cm)				
Crude	1.230 (0.075 to 2.386)	0.037*	0.026 (−0.864 to 0.916)	0.954
Adjusted for gestational age	0.659 (−0.373 to 1.690)	0.209	−0.422 (−1.208 to 0.364)	0.291
Fully adjusted^b^	0.802 (−0.337 to 1.942)	0.167	−0.241 (−1.129 to 0.648)	0.594

Chest circumference (cm)				
Crude	0.511 (−0.445 to 1.468)	0.293	0.035 (−0.695 to 0.766)	0.924
Adjusted for gestational age	0.013 (−0.827 to 0.852)	0.976	−0.346 (−0.984 to 0.292)	0.286
Fully adjusted^b^	0.166 (−0.740 to 1.072)	0.718	−0.014 (−0.717 to 0.690)	0.970

Head circumference (cm)				
Crude	−0.029 (−0.873 to 0.815)	0.947	−0.378 (−1.018 to 0.263)	0.246
Adjusted for gestational age	−0.255 (−1.082 to 0.571)	0.543	−0.555 (−1.180 to 0.070)	0.081
Fully adjusted^b^	0.166 (−0.306 to 0.638)	0.488	−0.093 (−0.783 to 0.597)	0.791

a Because PFOS and PFOA levels were log_10_-transformed, partial regression coefficients represent the expected change in dependent variables as a result of a 10-fold change in PFOS and PFOA levels.

b Adjusted for maternal age, maternal educational level, smoking status during pregnancy, maternal BMI, parity, gestational age, and blood sampling period. For head circumference, adjusted model also includes delivery mode (cesarean section/vaginal).

* Statistically significant, *p*-value < 0.05.

**Table 7:** Regression coefficients (95% CI) between PFOS and PFOA concentrations (ng/mL) in maternal serum and birth weight and birth size in female infants (*n* = 230).

Dependent variable	Partial regression coefficient of log_10_ PFOS^a^	*p*-Value	Partial regression coefficient of log_10_ PFOA^a^	*p*-Value
Birth weight (g)				
Crude	−90.7 (−308.6 to 127.1)	0.413	−35.9 (−207.7 to 136.0)	0.681
Adjusted for gestational age	−237.1 (−425.0 to −49.1)	0.014*	−107.8 (−256.1 to 40.4)	0.153
Fully adjusted^b^	−269.4 (−465.7 to −73.0)	0.007*	−76.7 (−234.7 to 81.3)	0.340

Length (cm)				
Crude	−0.224 (−1.237 to 0.788)	0.663	0.228 (−0.570 to 1.026)	0.574
Adjusted for gestational age	−0.855 (−1.752 to 0.042)	0.062	−0.080 (−0.787 to 0.626)	0.823
Fully adjusted^b^	−0.936 (−1.894 to 0.022)	0.055	−0.020 (−0.786 to 0.746)	0.959

Chest circumference (cm)				
Crude	−0.199 (−1.190 to 0.792)	0.693	−0.203 (−0.984 to 0.577)	0.608
Adjusted for gestational age	−0.774 (−1.670 to 0.121)	0.090	−0.490 (−1.191 to 0.212)	0.170
Fully adjusted^b^	−0.843 (−1.797 to 0.112)	0.083	−0.340 (−0.721 to 0.198)	0.263

Head circumference (cm)				
Crude	−0.227 (−0.957 to 0.504)	0.542	0.018 (−0.557 to 0.594)	0.950
Adjusted for gestational age	−0.443 (−1.165 to 0.278)	0.227	−0.086 (−0.652 to 0.479)	0.764
Fully adjusted^b^	−0.508 (−1.270 to 0.254)	0.191	0.001 (−0.605 to 0.608)	0.997

a Because PFOS and PFOA levels were log_10_-transformed, partial regression coefficients represent the expected change in dependent variables as a result of a 10-fold change in PFOS and PFOA levels.

b Adjusted for maternal age, maternal educational level, smoking status during pregnancy, maternal BMI, parity, gestational age, and blood sampling period. For head circumference, adjusted model also includes delivery mode (cesarean section/vaginal).

* Statistically significant, *p*-value < 0.05.
